# SOS-Independent Pyocin Production in P. aeruginosa Is Induced by XerC Recombinase Deficiency

**DOI:** 10.1128/mBio.02893-21

**Published:** 2021-11-23

**Authors:** Nina S. Baggett, Adam S. Bronson, Matthew T. Cabeen

**Affiliations:** a Department of Microbiology and Molecular Genetics, Oklahoma State University, Stillwater, Oklahoma, USA; Geisel School of Medicine at Dartmouth

**Keywords:** *Pseudomonas aeruginosa*, competition, heterogeneity, pyocins, recombinase

## Abstract

Pyocins are phage tail-like protein complexes that can be used by Pseudomonas aeruginosa to enact intraspecies competition by killing competing strains. The pyocin gene cluster also encodes holin and lysin enzymes that lyse producer cells to release the pyocins. The best-known inducers of pyocin production under laboratory conditions are DNA-damaging agents, including fluoroquinolone antibiotics, that activate the SOS response. Here, we report the discovery of an alternate, RecA-independent pathway of strong pyocin induction that is active in cells deficient for the tyrosine recombinase XerC. When Δ*xerC* cells were examined at the single-cell level, only a fraction of the cell population strongly expressed pyocins before explosively lysing, suggesting a that a built-in heterogenous response system protects the cell population from widespread lysis. Disabling the holin and lysin enzymes or deleting the entire pyocin gene cluster blocked explosive lysis and delayed but did not prevent the death of pyocin-producing cells, suggesting that Δ*xerC* cells activate other lysis pathways. Mutating XerC to abolish its recombinase activity induced pyocin expression to a lesser extent than the full deletion, suggesting that XerC has multiple functions with respect to pyocin activation. Our studies uncover a new pathway for pyocin production and highlight its response across a genetically identical population. Moreover, our finding that Δ*xerC* populations are hypersensitive to fluoroquinolones raises the intriguing possibility that XerC inhibition may potentiate the activity of these antibiotics against P. aeruginosa infections.

## INTRODUCTION

Pseudomonas aeruginosa is a Gram-negative bacterial species that is ubiquitous in the environment. It is also infamous as a multidrug-resistant human pathogen most frequently associated with infections in immunocompromised populations (1). This species is also known for aggressively competing with other bacterial cells using a variety of sophisticated offensive weapons. For instance, P. aeruginosa secretes soluble antibiotics like pyocyanin, enacts contact-mediated toxin injection using type VI secretion, and can produce phage tail-like bacteriocins (PTLBs) (2). Bacteriocins are typically named according to the producing species; in P. aeruginosa, they are termed pyocins and are thought to primarily enable intraspecies competition (3–5). There is also limited evidence that they may have activity against other species (6, 7). Pyocins are thought to allow cells to exploit ecological niches through competition and domination (5, 6).

Three types of pyocins are encoded by the P. aeruginosa genome; two of these, the R- and F-type pyocins, are PTLBs (2). The R-type pyocins are best understood with respect to their structure and mechanism; they are rod shaped and comprise a contractile sheath surrounding a core component with an iron atom-tipped spike (8). R-type pyocins kill closely related cells by binding to pyocin subtype-specific lipopolysaccharides on the surface of the target cell (9, 10) and contracting. The effectiveness of R-type pyocins at killing target cells has raised interest in using these complexes as precision antimicrobials (11–14). F-type pyocins differ from R-type pyocins in both structure and mechanism. This type of pyocin is a filamented flexible rod that enacts killing via a noncontractile mechanism (10, 15, 16). In P. aeruginosa strains PAO1 and PA14, the R- and F-type pyocins are encoded in a region between *trpE* and *trpG*, with the R-type genes first (17). The R/F pyocin gene cluster includes genes encoding a holin and a lysin (17), which function to perforate the cell membrane and digest the peptidoglycan cell wall, respectively, thereby releasing the relatively large R/F pyocins via lysis of producer cells.

The conditions that induce R/F pyocins in nature are not well understood, and the best-known inducer of pyocins is DNA-damaging agents, as pyocin expression is under the control of the SOS response (18, 19). The regulatory model includes a repressor, PrtR, which represses the expression of an activator protein, PrtN. DNA damage activates RecA, which binds to and stimulates autocleavage of PrtR, thereby derepressing *prtN*. PrtN subsequently binds to a conserved sequence, known as the P-box, at the promoter of the R/F pyocin gene cluster, leading to pyocin expression (20). Fluoroquinolone antibiotics such as ciprofloxacin are commonly used in antipseudomonal therapy. They stabilize DNA-protein (topoisomerase IV and DNA gyrase) intermediates, leading to double-strand breaks, likely via multiple mechanisms (21). Accordingly, fluoroquinolones induce the SOS response and stimulate pyocin production. Previous reports indicate that SOS-induced pyocin production increases the susceptibility of P. aeruginosa to fluoroquinolones because of cell lysis induced by the holin and lysin proteins (22–24). Hence, pyocin production imposes a cost on a population of bacterial cells and can sensitize strains to antibiotic treatment.

Costly behaviors, such as sacrificial cell lysis to release bacteriocins, can be managed via heterogeneity so that such behaviors are enacted by only some cells in a population. Such heterogeneity has been observed for colicin production by Escherichia coli (25) and has also been implicated in P. aeruginosa biofilms, where explosive lysis of a small subset of cells, mediated by the lysin gene of the R/F pyocin cluster, also functions to contribute extracellular DNA to biofilm communities (26). Microscopic observation of fluorescently tagged pyocin-like tailocins in Pseudomonas protegens also revealed heterogeneity in tailocin production (27). Similarly, P. aeruginosa virulence appears to benefit from the lysis of a subset of cells mediated by the Alp pathway. A holin-encoding operon, *alpBCDE*, is activated by AlpA when repression by AlpR (which is homologous to PrtR) is relieved under DNA damage conditions (28).

Here, we uncovered a previously unknown pathway for strong pyocin expression that is independent of RecA and unconnected to the SOS response. This pathway is active when levels of the recombinase XerC are lowered or when XerC recombinase activity is abrogated and is strongest when XerC is absent. Further, we analyzed Δ*xerC* strains at the single-cell level to visualize the distribution and dynamics of pyocin expression. We found striking heterogeneity, with a subset of cells progressively increasing pyocin expression before explosively lysing, implying a protective regulatory mechanism that commits only some cells to pyocin production and prevents widespread cell lysis.

## RESULTS

### Deletion of *PA14_69700* results in substantial upregulation of pyocin genes.

**FIG 1:**
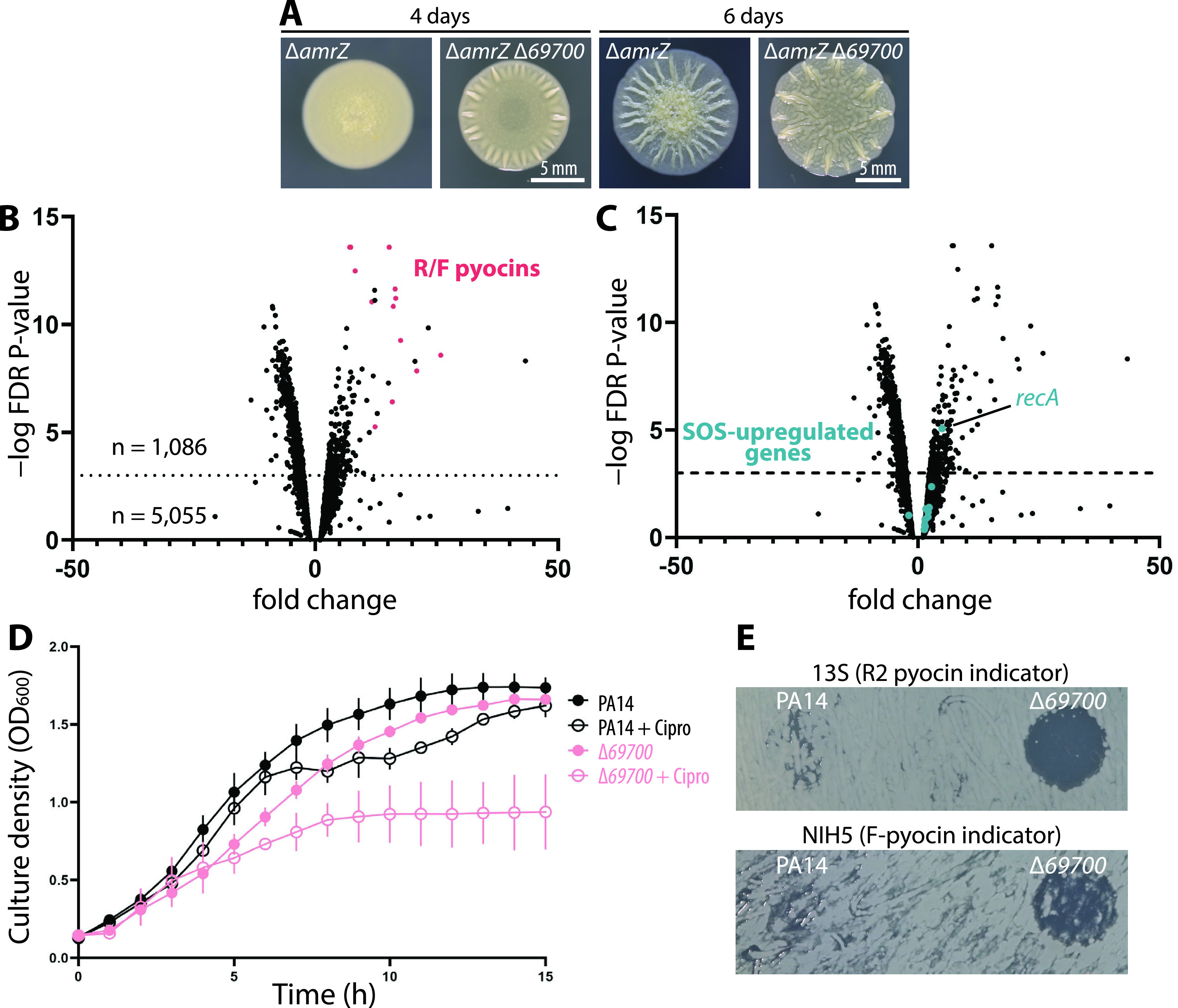
Pyocin genes, but not canonical SOS genes, are upregulated in a *Δ69700* strain, causing ciprofloxacin sensitivity. (A) Representative images of biofilm colonies grown on M6301 agar from which total RNA was extracted for transcriptomic analysis. RNA was extracted at day 4. (B) Volcano plot showing differentially regulated genes in a Δ*amrZ Δ69700* strain (MTC1398) versus the Δ*amrZ* parent (MTC590). A false-discovery rate (FDR) threshold of 10^−3^ was considered significant. R/F pyocin-encoding genes are shown in red. (C) The same volcano plot as in panel B, showing in teal genes that were strongly upregulated under SOS-inducing conditions in a study by Cirz et al. (19). (D) Representative growth curves of wild-type PA14 (MTC1) and Δ*69700* (MTC1513) strains in LB-Lennox in the absence and presence of 0.03 μg/mL ciprofloxacin. Error bars show standard deviations for at least three technical replicates. (E) Indicator assays showing R/F pyocin production by PA14 and the Δ*69700* mutant; clearing indicates the presence of pyocins in cell-free culture supernatants.

In previous work, we showed that an in-frame deletion of an uncharacterized gene, *PA14_69700* (also known as *PA14_RS28410*), resulted in enhanced biofilm formation by P. aeruginosa (29). To understand the mechanism by which *69700* deletion modulates biofilm formation, we performed transcriptomic analysis. We extracted RNA from *69700*^+^ and *Δ69700* biofilm colonies in the moderately hyperwrinkled Δ*amrZ* genetic background from which we first identified the *69700* gene (29). We anticipated finding one or more genes responsible for the hyperbiofilm phenotype of *69700* deletion strains (Fig. 1A). Deletion of *69700* resulted in a significant differential regulation of more than a sixth (1,062/6,142) of the entire P. aeruginosa PA14 genome (Fig. 1B). The differentially regulated genes are diverse in function: they include genes involved in metabolism, secretion systems (type II secretion system [T2SS] and T3SS), efflux, and transcriptional regulation and numerous genes whose functions remain unknown. However, there was a conspicuous group of adjacent, strongly upregulated genes (with log_2_ fold changes of ∼2 to 5) between *PA14_07970* and *PA14_08300*. These genes encode the R/F pyocins (Fig. 1B, red), which are phage tail-like, narrow-spectrum antimicrobial protein complexes produced by P. aeruginosa to kill closely related strains of the same species (2). Both the R and F types were highly upregulated. Given that deletion of *69700* increased the expression of R/F pyocins, we sought to characterize its regulatory role further.

### Hallmark SOS genes are not activated by *69700* deletion.

Canonical activation of the R/F pyocin gene cluster occurs following DNA damage and requires RecA activation, which also stimulates the SOS response (18–20). The SOS response regulates numerous genes, including genes involved in DNA repair and in metabolism. One trivial explanation for the observed pyocin gene upregulation is that the *69700* deletion activated the SOS response. However, among the set of known SOS-responsive genes in P. aeruginosa (19), none except *recA* exceeded our significance threshold (Fig. 1C). These data imply that *69700* deletion does not induce pyocin production by stimulating the SOS response, suggesting the existence of an alternate regulatory pathway for pyocin gene expression.

### The *69700* deletion mutant shows fluoroquinolone sensitivity and other pyocin-related phenotypes.

Previous reports suggested that pyocins are a determinant of sensitivity to fluoroquinolone antibiotics (22–24), prompting us to examine the fluoroquinolone sensitivity of the Δ*69700* mutant. We performed growth curve experiments in the presence and absence of subinhibitory concentrations of ciprofloxacin, which showed that both wild-type (WT) and Δ*69700* cells grew to an optical density at 600 nm (OD_600_) over 1.6. In the presence of ciprofloxacin (0.03 μg/mL), WT cells showed slower growth en route to a slightly lower final OD, while *Δ69700* cells reached an OD_600_ of only 0.8 (Fig. 1D), consistent with increased ciprofloxacin susceptibility correlating with elevated pyocin production in Δ*69700*. To confirm this observation, cell-free supernatants from WT and Δ*69700* strains were spotted onto lawns of P. aeruginosa indicator strains for R2 or F pyocins. Whereas supernatants from stationary WT cultures showed little inhibition (Fig. 1E), those from *Δ69700* cultures showed substantial inhibition of both indicator strains (Fig. 1E). Collectively, these experiments confirm that deletion of *69700* results in elevated R/F pyocin production.

### Pyocin-related phenotypes in a *69700* deletion are mediated by a deficit of XerC.

**FIG 2:**
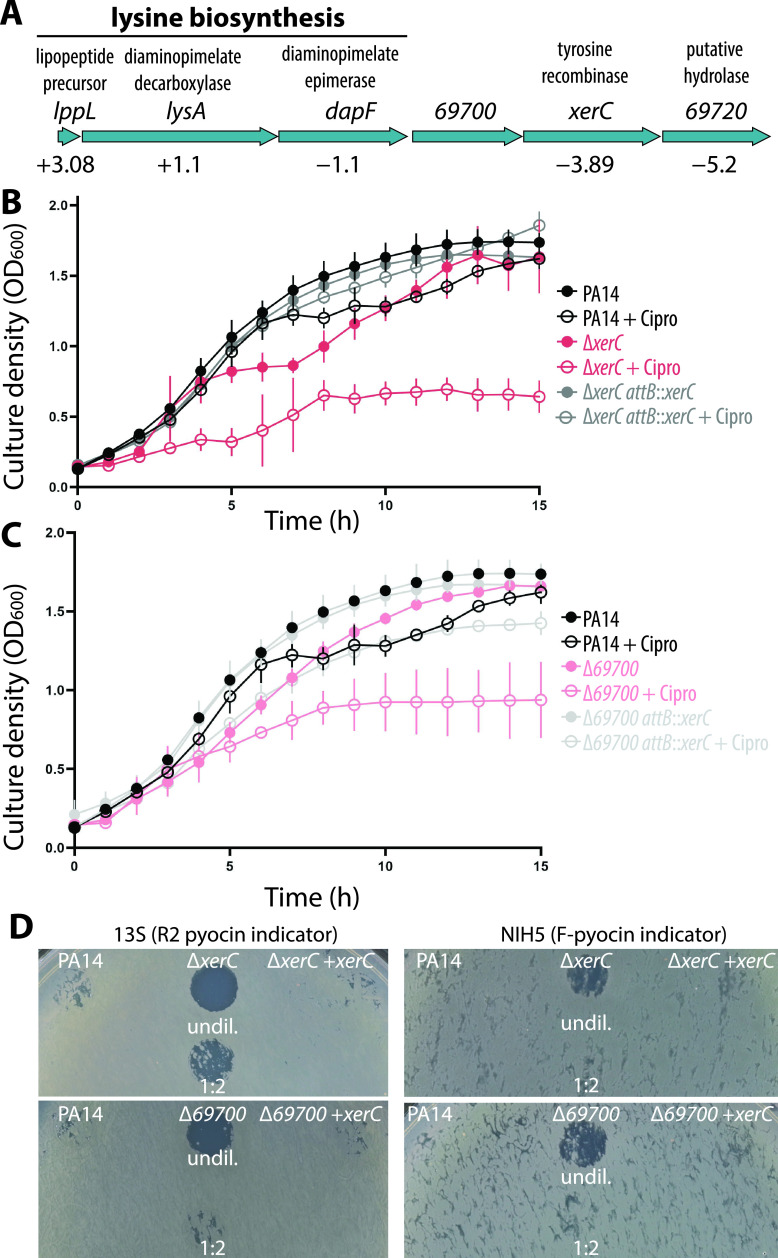
Pyocin gene upregulation and ciprofloxacin sensitivity in Δ*69700* is caused by deficiency of *xerC*. (A) Schematic of the *lppL* operon showing the encoded enzymes and their relative expression (fold change) in the Δ*amrZ* Δ*69700* strain (MTC1398) versus the Δ*amrZ* strain (MTC590). (B) Representative growth curves of PA14 (MTC1) and the Δ*xerC* (MTC2266) and Δ*xerC attB*::*CTX-1-P_lppL_-xerC* (“*attB*::*xerC*”) (MTC2262) mutants in LB-Lennox medium with and without 0.03 μg/mL ciprofloxacin. (C) Representative growth curves of PA14 and the Δ*69700* (MTC1513) and Δ*69700 attB*::*CTX-1-P_lppL_-xerC* (“*attB*::*xerC*”) (MTC2264) strains in LB-Lennox medium with and without 0.03 μg/mL ciprofloxacin. Error bars in panels B and C show standard deviations for at least three technical replicates. (D) Indicator assays showing R/F pyocin production by PA14 compared to Δ*69700* and Δ*xerC* strains complemented or not with *xerC* (*attB*::*CTX-1-P_lppL_-xerC*). The supernatants were diluted 2-fold with fresh sterile LB where indicated.

**FIG 3:**
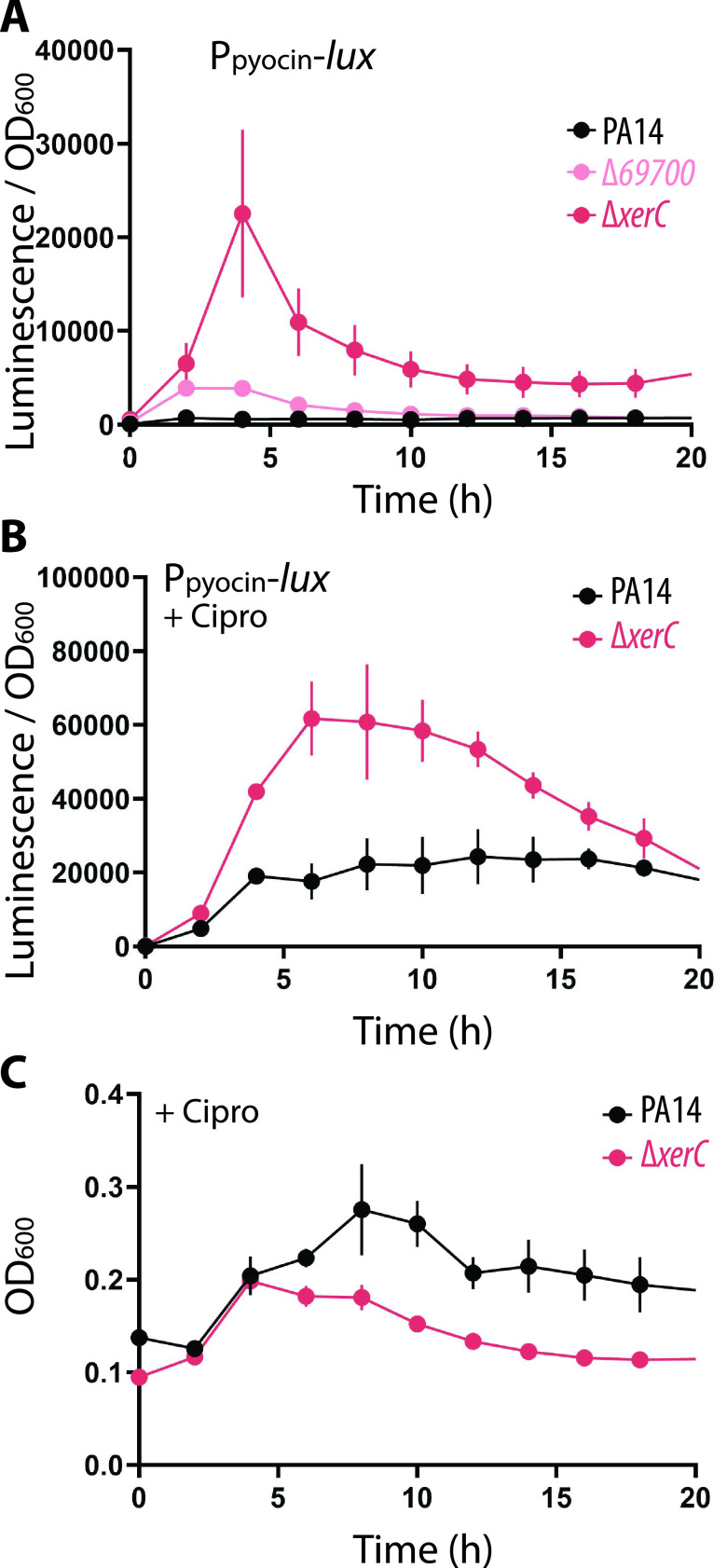
Deletion of *xerC* increases pyocin gene expression. (A) Representative transcriptional profile of a P*_07990_-lux* reporter (reporting on R/F pyocin gene transcription) during growth in LB-Lennox by wild-type PA14 (MTC2280), Δ*69700* (MTC2281), or Δ*xerC* (MTC2297) strains. (B) Representative transcriptional profile as in panel B of wild-type PA14 or Δ*xerC* strains treated with 0.06 μg/mL ciprofloxacin. (C) Growth curves from the experiment reported in panel B. Error bars show standard deviations for at least three technical replicates; some error bars are smaller than the graph symbols.

The transcriptomic data revealed that the two genes downstream of *69700* in its operon, *xerC* and *69720*, were downregulated despite our use of a markerless, in-frame deletion of *69700* (Fig. 2A). Since *xerC* (previously called *sss*) encodes a tyrosine recombinase enzyme (30, 31), we determined whether *xerC* contributed to elevated pyocin production. When a *xerC* deletion strain was challenged with ciprofloxacin it exhibited a sensitivity to ciprofloxacin similar to Δ*69700* (Fig. 2B). Its sensitivity could be reversed by chromosomal complementation of *xerC* (driven by the operon promoter) at the *attB* locus (Fig. 2B). Importantly, the ciprofloxacin sensitivity of a Δ*69700* strain was also reversed by chromosomal complementation with *xerC* (Fig. 2C), strongly suggesting that decreased *xerC* expression is responsible for the ciprofloxacin sensitivity phenotype of Δ*69700* cells. The supernatants from both Δ*xerC* and Δ*69700* strains exhibited significant clearing on the indicator strain, whereas no clearing was observed from either strain complemented with *xerC* (Fig. 2D). These experiments show that increased pyocin production in Δ*69700* can be attributed to downregulation of *xerC*, so that a Δ*69700* deletion essentially acts as a hypomorphic allele of *xerC*. We therefore used Δ*xerC* strains for further characterization of the pyocin overproduction phenotype. We quantified pyocin expression by chemiluminescence, placing a luciferase reporter under the control of the R/F pyocin promoter. This system revealed that the *xerC* deletion increased R/F pyocin expression approximately 5-fold more than the *69700* deletion (Fig. 3A), consistent with the idea above that *69700* deletion resembles a hypomorphic *xerC* allele. Furthermore, treatment of Δ*xerC* cells with sublethal (0.03 μg/mL) ciprofloxacin substantially increased pyocin expression approximately 3-fold above that in treated WT cells (Fig. 3B). This result argues that *xerC* deficiency not only raises the basal level of pyocin expression but also magnifies antibiotic-induced pyocin production, which may explain the increased susceptibility of Δ*xerC* cells to ciprofloxacin (Fig. 2B and 3C).

### Pyocin production in the *xerC* deletion requires PrtN but not RecA.

**FIG 4:**
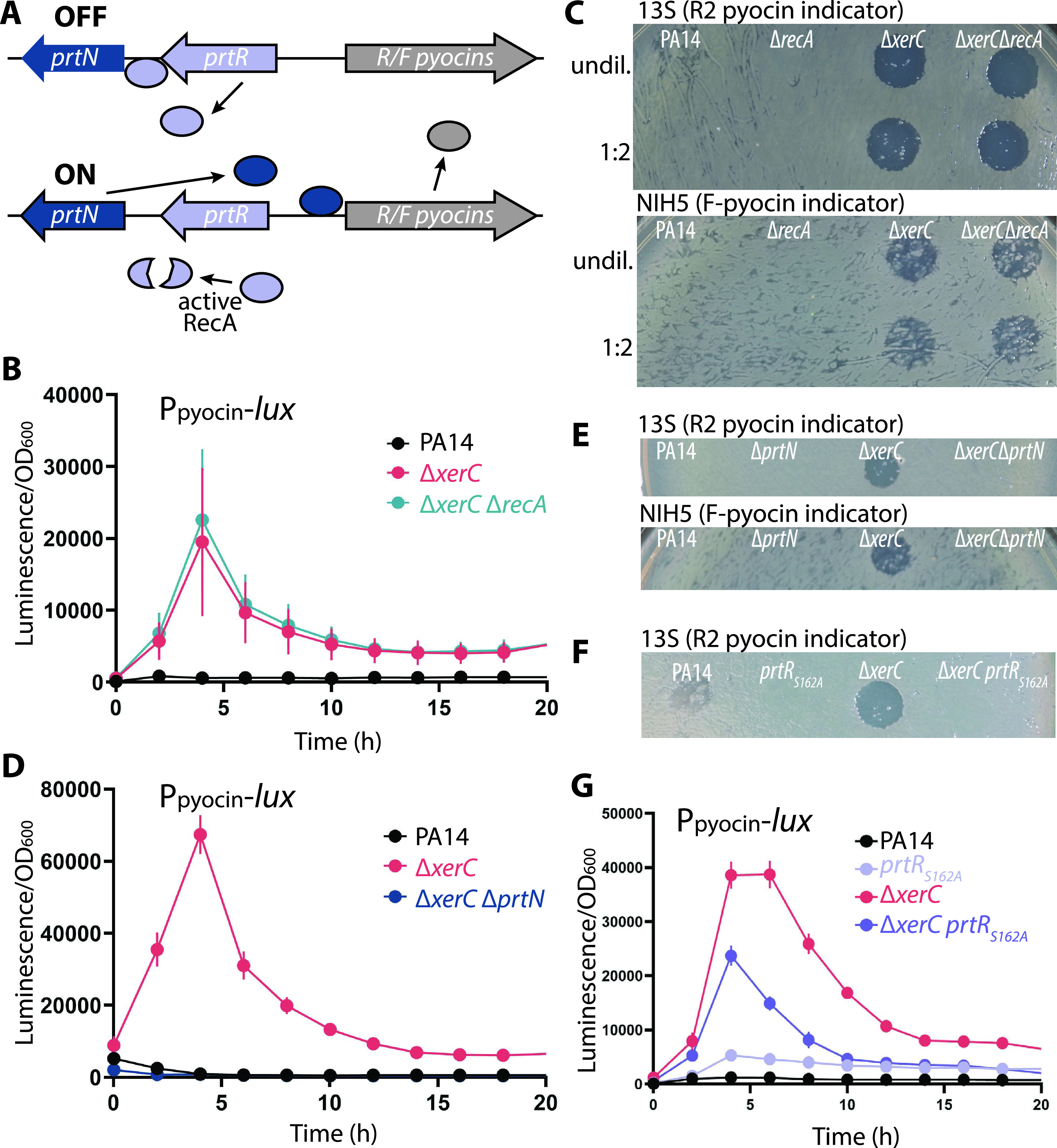
Expression and production of pyocins by Δ*xerC* does not require RecA but requires PrtN. (A) Schematic of RecA-PrtN/R-mediated pyocin production. When the SOS response is inactive, PrtR represses *prtN* transcription so that pyocin expression is off. The presence of activated RecA during the SOS response induces PrtR autoproteolytic cleavage, permitting PrtN production and hence activating pyocin gene expression. (B) Representative transcriptional profiles (P*_07990_-lux* reporter) of PA14 (MTC2280), Δ*xerC* (MTC2297), and Δ*xerC* Δ*recA* (MTC2301) strains. (C) Pyocin indicator assays using cell-free stationary-phase culture supernatants from PA14, Δ*recA* (MTC2274), Δ*xerC* (MTC2266), and Δ*xerC* Δ*recA* (MTC2288) strains. The supernatants were diluted 2-fold with fresh sterile LB where indicated. (D) Representative transcriptional profiles (P*_07990_-lux* reporter) of PA14, Δ*xerC*, and Δ*xerC* Δ*prtN* (MTC2298) strains. (E) Pyocin indicator assays as indicated using cell-free stationary-phase culture supernatants from PA14, Δ*prtN* (MTC2276), Δ*xerC* (MTC2266), and Δ*xerC* Δ*prtN* (MTC2289) strains. (F) Pyocin indicator assay using cell-free stationary-phase culture supernatants of PA14, the Δ*xerC* mutant, and their counterparts producing uncleavable PrtR_S162A_ as the only source of PrtR in the cell (MTC2305 and MTC2304, respectively). (G) Representative transcriptional profiles (P*_07990_-lux* reporter) of PA14 and Δ*xerC* strains without or with (MTC2308 and MTC2307, respectively) uncleavable PrtR_S162A_ as the only source of PrtR in the cell. Error bars in panels B, D, and G show standard deviations for three technical replicates.

We next explored the basis by which *xerC* deletion induces pyocin expression. The known genetic mechanism for the expression of R-type pyocins requires the RecA protein in combination with a repressor, PrtR, and an activator, PrtN (20). Under uninduced conditions, PrtR binds to an operator in the *prtN* promoter and represses its expression (Fig. 4A). Activated RecA (i.e., when DNA damage is sensed) stimulates PrtR autocleavage, thereby derepressing *prtN*. PrtN then binds the promoter of the pyocin region at putative P-box regions, inducing expression of the R/F pyocin genes (Fig. 4A) (17, 20). We therefore determined if pyocin expression was dependent on *recA* in the *ΔxerC* mutant. We observed the expected low levels of pyocin expression in WT cultures, whereas similar, highly elevated pyocin expression (via luciferase-stimulated chemiluminescence) (Fig. 4B) and killing activity (Fig. 4C) were observed for both Δ*xerC* and Δ*xerC* Δ*recA* mutants. Collectively, these data show that production of pyocins in the absence of *xerC* occurs independently of RecA, thus bypassing a key element of the canonical regulatory mechanism for pyocin expression.

These results suggested that pyocin expression might also be independent of the pyocin gene activator PrtN, which was tested by deleting *prtN* in a Δ*xerC* background. Surprisingly, little to no pyocin expression or killing activity was observed in Δ*xerC* Δ*prtN* cultures (Fig. 4D and E, respectively), indicating that pyocin expression in Δ*xerC* strains requires PrtN. We then tested whether PrtR autocleavage is required by introducing a gene encoding an uncleavable PrtR variant (S162A) (23) at the native chromosomal locus. In both wild-type and Δ*xerC* strains, the uncleavable PrtR variant abolished detectable pyocin killing activity (Fig. 4F), whereas pyocin gene expression was diminished but not fully abolished (Fig. 4G). Thus, pyocin expression in *xerC* deletion strains is strictly dependent on PrtN and at least partially repressible by PrtR. These results suggest the existence of a separate and previously unknown regulatory pathway for pyocins that bypasses RecA and is abetted by but not fully dependent on PrtR cleavage to relieve *prtN* repression.

### Pyocin expression is highly heterogeneous among individual cells.

Elevated pyocin production, whether stimulated by DNA damage or induced by XerC deficiency, poses a challenge to cells. The holin and lysin enzymes encoded as part of the R/F pyocin cluster (17) were substantially upregulated (approximately 8- and 16-fold, respectively) by *69700* deletion in our transcriptomic data. Upregulation of these lytic enzymes would presumably impose a fitness cost, as widespread cell lysis would severely hamper population growth. One means of managing costly phenotypes is through heterogeneity, wherein only some cells in a population engage in behaviors that are costly or deleterious but that benefit the remainder of the population. In support of this idea, two previous reports suggested that pyocins are heterogeneously expressed. First, in P. aeruginosa biofilms, a small subset of cells was observed to explosively lyse and release their DNA, and this lysis was dependent on the lysin encoded in the R/F pyocin gene cluster (26). Moreover, a green fluorescent protein (GFP) transcriptional reporter for the holin-encoding gene in the pyocin cluster was active in only a subset of cells (26). More recently, a study in P. protegens examined fluorescent fusions to tailocins, which are similar to pyocins, to directly observe intraspecies killing. In that work, tailocin production was likewise observed to be heterogeneous (27). Building on this evidence, we thus hypothesized that pyocin expression would be heterogeneous, even when strongly induced by *xerC* deletion. We tested this hypothesis by visualizing pyocin expression at the single-cell level, constructing a GFP reporter for pyocin gene expression and observing cells using fluorescence microscopy.

**FIG 5:**
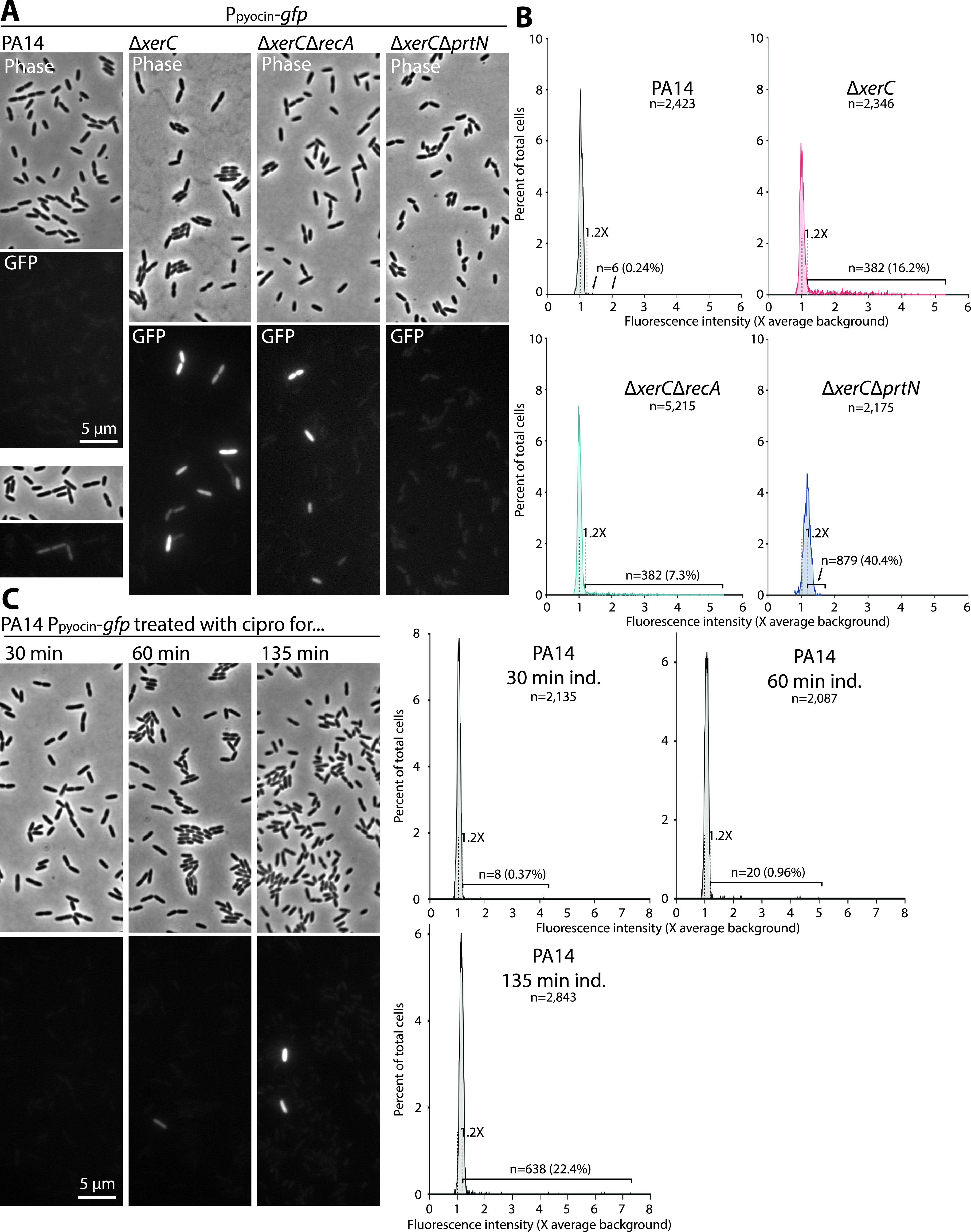
Pyocin expression is heterogeneous across individual cells. (A) Representative phase-contrast and fluorescence micrographs of PA14 (MTC2277), Δ*xerC* (MTC2252), Δ*xerC* Δ*recA* (MTC2291), and Δ*xerC* Δ*prtN* (MTC2292) cells growing on agarose pads. (B) Line histograms of average GFP fluorescence in individual cells of the indicated strains. Fluorescence is plotted as a multiple of the average background value, which is indicated with a black dashed line. The gray dashed line represents 1.2× the average background value, which was set as the threshold for GFP positivity (see Materials and Methods and Fig. S2). The numbers and percentages of positive cells are indicated. (C) Representative micrographs of PA14 pyocin-reporter cells treated in liquid culture for the indicated times with 1 μg/mL ciprofloxacin. (D) Line histograms of GFP mean fluorescence in individual cells of the indicated strains. Annotations are as in panel B.

In wild-type cells, which showed very little pyocin gene expression in bulk assays, we saw very few GFP-producing cells (0.24%) that were relatively dimly fluorescent (Fig. 5A and B). In contrast, Δ*xerC* cells, while mostly GFP negative, showed a much greater proportion of GFP-positive cells (16.2%) that varied in brightness but were generally much brighter than in the wild type. In a Δ*xerC* background, deletion of *recA* lowered the fraction of GFP-positive cells to 7.3%, consistent with the canonical RecA-PrtR pathway partially contributing to pyocin expression in Δ*xerC* cells. Deletion of *prtN* fully abolished the appearance of bright GFP-positive cells (Fig. 5A and B), in accord with the bulk data (Fig. 4B to D), but the overall fluorescence distribution was shifted so that some 40% of cells fell just above our cutoff for GFP positivity (Fig. 5B). Even in Δ*xerC* backgrounds, the majority of cells displayed little to no detectable GFP expression, supporting our hypothesis that a relatively small subset of cells strongly produces pyocins and is sacrificed for the fitness of the entire community.

10.1128/mBio.02893-21.2 FIG S2 Determination of GFP fluorescence cutoff for positive cells. Line histogram of average per-cell fluorescence of PA14 cells containing no GFP reporter (MTC1) and imaged in the GFP channel as with our GFP reporter. The fluorescence of 100% of reporterless cells fell within 5 standard deviations of the mean value, and we therefore chose 1.2× the background value, which is between 5 and 6 standard deviations above the mean, as the cutoff for designating cells GFP positive. Download FIG S2, PDF file, 0.04 MB. Copyright © 2021 Baggett et al. 2021 Baggett et al. https://creativecommons.org/licenses/by/4.0/ This content is distributed under the terms of the Creative Commons Attribution 4.0 International license.

We also examined the DNA damage-induced pathway using a 1-μg/mL concentration of ciprofloxacin that was previously used to induce SOS in P. aeruginosa (19). While we observed more pyocin-expressing cells in response to SOS induction, the proportion of bright GFP-expressing cells under the observed treatment durations was much lower than for Δ*xerC* cells (Fig. 5C), in accord with bulk assays (Fig. 3B and C). Interestingly, after 135 min of induction, we observed a slight shift in overall fluorescence, as in the Δ*xerC* Δ*prtN* strain, so that many cells fell at the bottom end of our GFP-positive range. Irrespective of the mode of induction, genetic or via drugs, population heterogeneity appears to be a hallmark of pyocin gene expression, suggesting the existence of a regulatory mechanism that introduces heterogeneity, even in an isogenic population, to limit sacrificial production of pyocins to only a few cells.

### Loss of XerC recombinase activity modestly increases pyocin expression.

**FIG 6:**
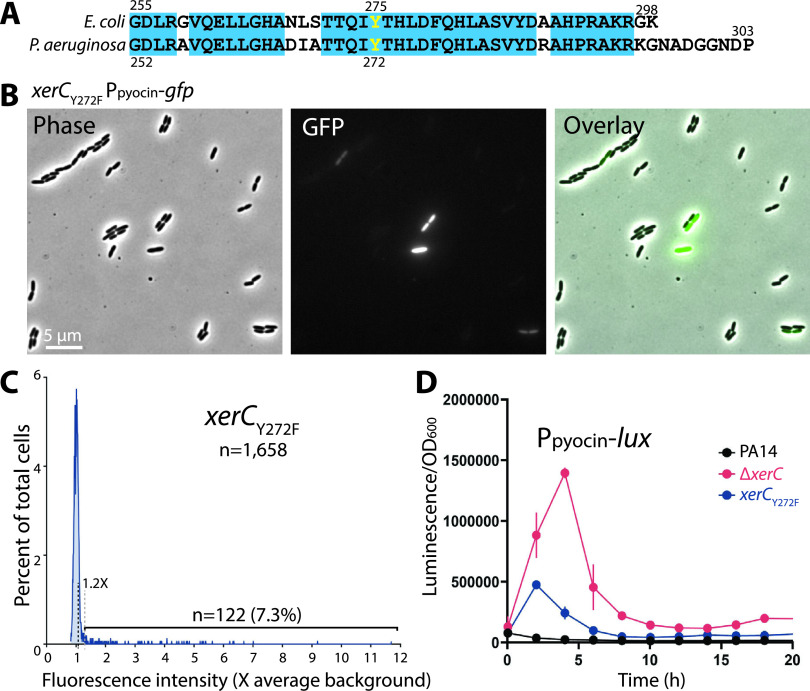
Effect of XerC recombinase inactivation. (A) Sequence alignment of the C-terminal ends of E. coli MG1655 and P. aeruginosa PA14 XerC proteins showing conservation (cyan boxes) and the catalytic Tyr residue (yellow). (B) Representative phase-contrast and fluorescence micrographs of PA14 *xerC*_Y272F_ bearing a P*_07990_*-*gfp* reporter at *attB* (MTC2341). (C) Line histograms of GFP mean fluorescence in individual PA14 *xerC*_Y272F_ cells (MTC2341). (D) Representative transcriptional profiles (P*_07990_-lux* reporter) of PA14 *xerC*_Y272F_ cells (MTC2339). Error bars show standard deviations for three technical replicates; some error bars are smaller than the symbols.

The increased levels of pyocin expression in Δ*xerC* strains, whether observed in bulk or at the single-cell level, made us ask whether it was specifically loss of XerC recombinase activity, or some other function of XerC, that induced pyocins. We thus constructed a recombinase-dead XerC_Y272F_ point mutant, in analogy to the catalytically inactive Escherichia coli XerC_Y275F_ mutant (32). The catalytic Tyr residue is located in a region of high sequence conservation between E. coli and P. aeruginosa (Fig. 6A). Qualitative microscopic observation of *xerC*_Y272F_ cells bearing a GFP reporter for pyocin expression (Fig. 6B) gave the impression that there were more pyocin-ON cells than in the wild type, but not as many as in a *xerC* deletion. Quantitation of the fluorescence data (Fig. 6C) and bulk measurements with the pyocin luciferase reporter (Fig. 6D) indeed showed an intermediate phenotype for the point mutant compared with wild-type or Δ*xerC* cells. These data argue that elevated pyocin expression in Δ*xerC* strains is not due solely to loss of XerC recombinase activity, implying another function of XerC with respect to pyocin expression.

### Pyocin expression is a terminal phenotype.

**FIG 7:**
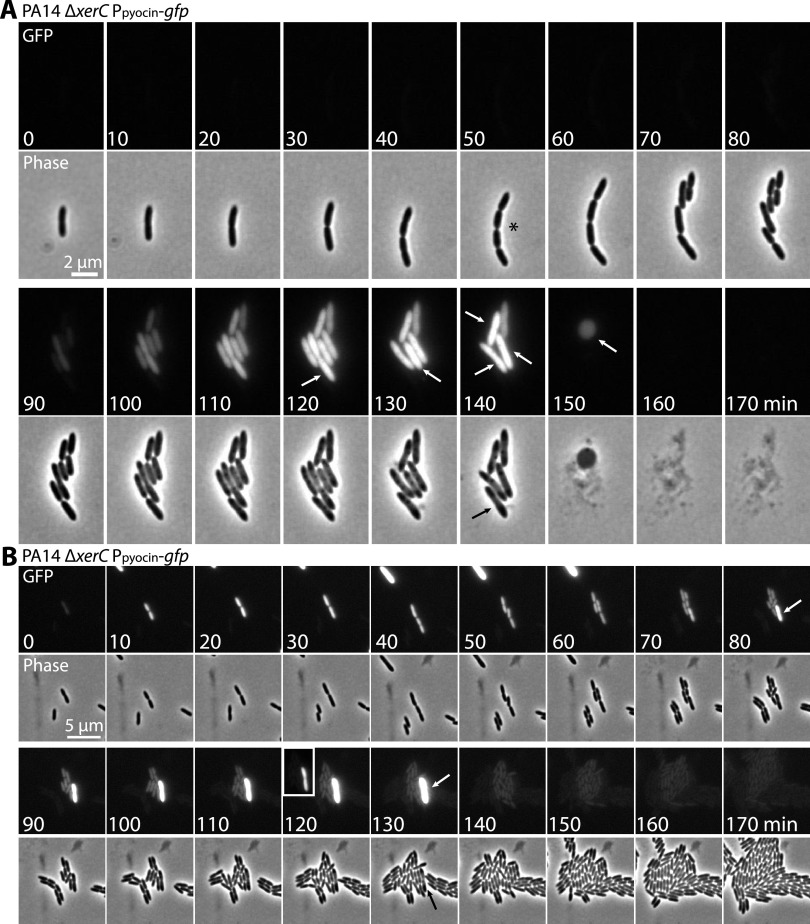
Fate of cells that turn on pyocin expression. (A) Time-lapse series of fluorescence (GFP) and phase micrographs of PA14 Δ*xerC* cells bearing a P*_07990_*-*gfp* reporter at *attB* (MTC2252) to report on R/F pyocin expression. Asterisk indicates chained cells. White arrows denote cells that lysed before the next time point. Black arrow indicates a cell that showed no GFP expression but that lysed with the other cells in the microcolony. (B) Time-lapse series as in panel A showing both a cell that strongly expressed GFP and lysed (white arrows) and cells that initially weakly expressed GFP but then appeared to turn off pyocin expression and continue growing. Inset (120 min): Image rescaled to show the relative weakness of GFP expression in cells that turned pyocin expression off compared to the strong expression of the cell that lysed.

The heterogeneity of pyocin gene expression under every condition examined prompted us to expand on our results and previous work (26, 27) by tracking the expression levels and fates of pyocin-producing cells using time-lapse fluorescence imaging. We predicted that pyocin expression would result in cell death and/or lysis due to holin and lysin activity. We used a Δ*xerC* background because of the relatively frequent appearance of GFP-positive, pyocin-expressing cells in this strain (Fig. 5A). Time-lapse microscopy revealed several phenotypes. First, we occasionally observed modest cell chaining, with groups of 4 to 8 cells appearing to be incompletely separated after cell duplication, in the Δ*xerC* background (Fig. 7A, asterisk) that was short-lived and was dissimilar from the cellular filamentation or doublets reported for XerC-deficient E. coli (33, 34). This suggests that loss of XerC has a less severe effect on cell division and chromosomal partitioning in P. aeruginosa than in E. coli. Second, cells progressively increased in GFP reporter intensity until they suddenly and explosively lysed, leaving behind visible debris (Fig. 7A). Third, we occasionally observed spherical cells or spheroplasts as an intermediate step in cell lysis (Fig. 7A, 150 min), consistent with previous observations (26). Fourth, we frequently observed that a cluster of sister cells would all express the GFP reporter at about the same time (Fig. 7A), suggesting that the initiating event for pyocin expression occurs a few cell divisions before we can detect GFP production. Fifth, we occasionally observed a cell without strong GFP expression that nonetheless lysed (Fig. 7A, 140 min, black arrow). Finally, pyocin production appeared to be a terminal phenotype, as nearly every cell we observed expressing GFP later showed explosive lysis. We did rarely observe cells that appeared to turn off pyocin-GFP reporter expression after expressing some GFP (Fig. 7B), suggesting that pyocin expression is reversible. However, in such cases, the peak GFP fluorescence was much lower than in cells that went on to lyse (compare the bright cell in Fig. 7B, 80 min and following, with the relatively dim surrounding cells). Collectively, these results suggest that a subset of cells commits to pyocin expression and then strongly expresses pyocins until lysis.

### Disabling the holin and lysin genes delays but does not prevent cell death.

**FIG 8:**
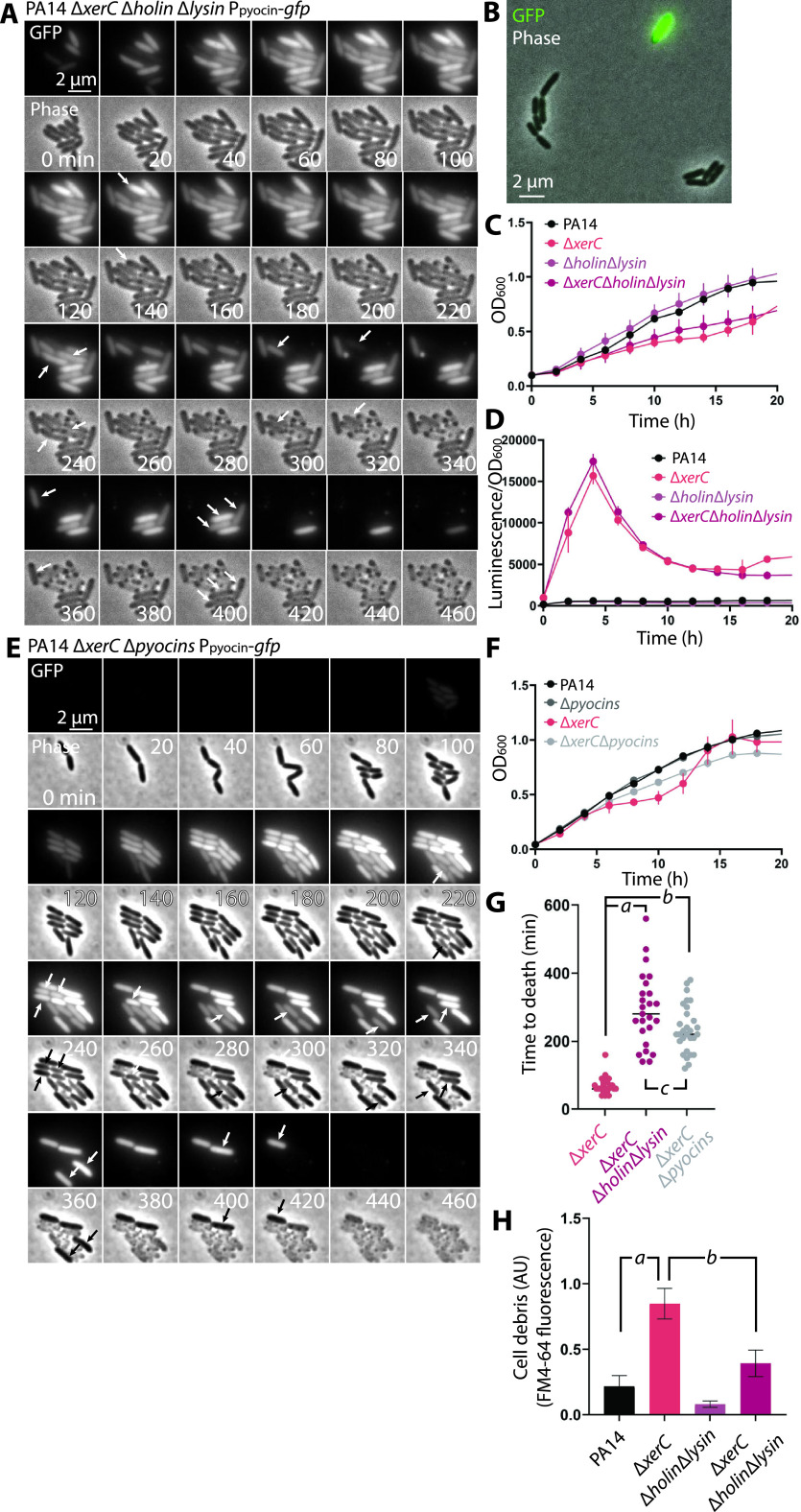
Fate and phenotypes of pyocin-expressing cells with deletions of lysis genes. (A) Time-lapse series of fluorescence (GFP) and phase micrographs of PA14 Δ*xerC* Δ*holin* Δ*lysin* cells bearing a P*_07990_*-*gfp* reporter at *attB* (MTC2293) to report on R/F pyocin expression. Arrows denote apparent cell death accompanied by loss of GFP fluorescence. (B) Representative GFP-phase contrast overlay of MTC2293 cells in exponential phase. (C) Representative growth curves of wild-type PA14 (MTC2280), Δ*xerC* (MTC2297), Δ*holin* Δ*lysin* (MTC2284), and Δ*xerC* Δ*holin* Δ*lysin* (MTC2299) strains grown in LB-Lennox medium. (D) Representative transcriptional profiles (P*_07990_-lux* reporter) of the same experiment as in panel C. (E) Time-lapse series of fluorescence (GFP) and phase micrographs of PA14 Δ*xerC* Δ*pyocins* cells bearing a P*_07990_*-*gfp* reporter at *attB* (MTC2332) to report on R/F pyocin expression. Arrows denote apparent cell death accompanied by loss of GFP fluorescence. (F) Representative growth curves of wild-type PA14 (MTC1), Δ*xerC* (MTC2266), Δ*pyocins* (MTC2326) and Δ*xerC* Δ*pyocins* (MTC2324) cells grown in LB-Lennox medium. (G) Manually curated plot of time intervals between appearance of visible GFP fluorescence and cell lysis or death (loss of GFP fluorescence) in Δ*xerC* (MTC 2252), Δ*xerC* Δ*holin* Δ*lysin* (MTC2293), and Δ*xerC* Δ*pyocins* (MTC2324) cells (*n* = 25 for each condition). Horizontal lines indicate the mean value for each condition. Italic letters denote *P* values (2-tailed Student's *t* test): *a*, 2.9 × 10^−10^; *b*, 2.0 × 10^−12^; *c*, 0.03. (H) Graph of cell debris produced by PA14 (MTC1), Δ*xerC* (MTC2266), Δ*holin* Δ*lysin* (MTC2295), and Δ*xerC* Δ*holin* Δ*lysin* (MTC2294) cells, as assayed by ultracentrifugation and FM4-64 staining of cell-free supernatants. Values shown are averages from three separate experiments, with error bars denoting standard deviations. Italic letters denote *P* values (2-tailed Student's *t* test): *a*, 0.0018; *b*, 0.0056. Error bars in panels C to F indicate standard deviations for at least 3 technical replicates; some bars are smaller than the graph symbols.

The striking explosive lysis of pyocin-producing cells was consistent with the long-standing model that holin and lysin enzymes are involved in pyocin release. To more formally test this model, we deleted the holin- and lysin-encoding genes from the pyocin gene cluster and observed pyocin reporter cells in bulk and microscopically. In Δ*xerC* cells with deletions of holin and lysin, we observed heterogeneous expression of GFP that was qualitatively similar to our observations in the Δ*xerC* strain (Fig. 8B). In bulk, the Δ*xerC* strain with deletions of the holin and lysin genes (referred to here as the Δ*xerC* Δ*holin* Δ*lysin* strain) showed slightly faster growth (Fig. 8C) and greater luciferase expression (Fig. 8D) than a Δ*xerC* strain. Surprisingly, time-lapse imaging (Fig. 8A) revealed that pyocin-expressing cells still died, even in the absence of the holin and lysin, but with at least two key differences from strains encoding the holin and lysin. First, the time between detectable pyocin-GFP reporter expression and cell death in a strain lacking the holin and lysin genes was significantly delayed relative to a strain containing these genes (Fig. 8G). This longer delay between pyocin expression and cell death is a likely explanation for the faster cell growth and greater pyocin expression observed in bulk (Fig. 8C and D). Second, rather than explosively lysing, pyocin-expressing cells appeared to “deflate,” losing their GFP fluorescence and becoming brighter in phase-contrast images (Fig. 8A). In accord with this distinct mode of death, cells with deletion of the holin and lysin also showed much less cell debris in culture supernatants than their holin- and lysin-replete counterparts (Fig. 8H).

### R/F pyocin production is not fully responsible for cell death of pyocin-ON cells.

The nonexplosive death of pyocin-ON holin-lysin mutant cells raised the question of whether other proteins encoded by the R/F pyocin gene cluster might mediate cell death. Thus, we constructed a full deletion of the entire R/F pyocin gene cluster and tracked the fate of pyocin-ON cells (those activating the GFP transcriptional reporter under the control of the pyocin promoter). Even with the full pyocin deletion, these cells nonetheless showed a death phenotype that mimicked that of the holin-lysin mutant (Fig. 8E), with slightly slower growth (Fig. 8F) and a similar time to death (Fig. 8G). This phenotype implies the existence of one or more factors that lead to cell death and are induced via the same pathway as R/F pyocins—perhaps under the control of PrtN.

## DISCUSSION

For years, the only known inducer of pyocin expression, and even the expression of unrelated bacteriocins in other species, including E. coli, was DNA damage and the SOS response (10, 35–38). In this work, we discovered a new pathway for pyocin production in P. aeruginosa cells that is both independent of and stronger than the SOS-induced pathway. This previously unknown pathway is induced by a lack of the XerC tyrosine recombinase. We show that pyocin production is strongly heterogeneous across a cell population and that pyocin-ON cells display progressively strengthening expression before lysis or death. We speculate that the genetic circuitry underlying pyocin expression ensures that only some cells in a population commit to sacrificial cell lysis.

The more modest pyocin upregulation in a Δ*69700* strain than in a Δ*xerC* deletion strain suggests that the alternative pathway for pyocin induction scales with the degree of XerC deficiency, with its complete absence causing a stronger phenotype than its downregulation. XerC has not been extensively studied in P. aeruginosa. The modest chaining phenotype observed in the Δ*xerC* deletion is consistent with a role in chromosome separation, but a less critical role than in Escherichia coli (33, 34). We do not yet fully understand how absence of XerC causes pyocin gene upregulation, but its RecA independence and the lack of SOS gene upregulation in a Δ*69700* deletion strain argue against DNA damage or SOS responses as an initiating factor. Still, we cannot rule out at least some contribution of RecA-dependent processes to pyocin expression in Δ*xerC* cells, as *recA* deletion reduced the proportion of GFP-positive cells in our analyses (although it did not substantially change observable pyocin expression in bulk). A partial dependence on RecA would be consistent with previous E. coli work showing at least some SOS-induced cells in a Δ*xerC* background (39).

The less severe phenotype of the XerC_Y272F_ recombinase-dead mutant compared to the full deletion suggests that XerC may have multiple functions in regulating pyocin expression. It will be interesting to learn whether deletion of other recombinases, such as XerD, similarly elicits pyocin production. The findings that pyocin upregulation in Δ*xerC* can occur independently of RecA, is partially repressed by PrtR, and is dependent on PrtN demonstrates that PrtR cleavage and *prtN* derepression can occur by a previously unknown mechanism that is independent of the SOS response. Previous work has shown that certain other mutations, such as in the oligoribonuclease-encoding *orn* gene, upregulate pyocin expression but in a RecA/SOS-dependent manner (40). More recently, inactivation of *fis* (factor for inversion stimulation) increased pyocin production by increasing *prtN* expression by approximately 2.7-fold; Fis was shown to directly bind to the *prtN* promoter as an apparent repressor (41). This effect appeared to be independent of the SOS response (41) but was substantially weaker than the impact of reduced XerC levels, as *prtN* was upregulated approximately 10-fold, even in the Δ*69700* strain we used for our transcriptomic studies. Hence, the phenotype of Δ*xerC* is distinguished both by its SOS independence and by its strong *prtN* and pyocin expression.

Our time-lapse microscopy shows that pyocin expression is rarely “turned off” once activated, with the vast majority of pyocin-positive cells growing brighter and brighter until cell lysis or death. Interestingly, we always observed strong heterogeneity, with individual cells typically being strongly on or completely off. We hypothesize that positive autofeedback in *prtN* expression may be involved in this pattern, as it would be one straightforward mechanism to explain the observed heterogeneity. We note that heterogeneous responses of individual bacterial cells to a particular internal or environmental condition are common, with different underlying mechanisms (42–45).

We were initially surprised to find that pyocin-producing cells died even in the absence of the holin and lysin or in the absence of the entire R/F pyocin gene cluster. Although these cells do not explosively lyse, the loss of GFP fluorescence suggests that the integrity of the cell envelope is compromised. What kills these cells? It is tempting to speculate that one or more other lytic proteins are also under the control of PrtN. The AlpBCDE proteins, which include phage holin-like enzymes that can cause cell lysis (28), are strong candidates, as *alpB* was upregulated approximately 6-fold in our Δ*69700* transcriptomic data. Activation by PrtN of the *alpBCDE* cluster, typically activated by its own activator, AlpA (28, 46), would be particularly interesting, with such activator cross talk perhaps serving as a fail-safe to ensure cell lysis when pyocins are expressed. Another possibility is that the absence of XerC may independently induce the Alp system, in accord with our observation of occasional cell lysis without strong pyocin expression (Fig. 7A). Another lytic protein, CidA (47), was downregulated approximately 2-fold in our transcriptomic data and so we consider it a less likely candidate.

We note briefly that our experiments identifying *xerC* as the critical gene for the pyocin phenotype also hinted at a potential role for *xerC* in biofilm formation, at least as assessed by colony morphology (Fig. S1). The failure of *xerC* complementation to reverse the wrinkled morphology of a Δ*69700* strain (Fig. S1) suggests that *69700* is itself a determinant of biofilm formation, independent of the effects of *69700* deletion on *xerC* expression.

10.1128/mBio.02893-21.1 FIG S1 Effects of *xerC* deletion and complementation on colony morphology. Colony morphology after 6 days of growth on M6301 agar of the indicated strains. Wrinkled morphology is typically associated with biofilm formation. Download FIG S1, PDF file, 0.1 MB. Copyright © 2021 Baggett et al. 2021 Baggett et al. https://creativecommons.org/licenses/by/4.0/ This content is distributed under the terms of the Creative Commons Attribution 4.0 International license.

Finally, our results showed a striking effect of ciprofloxacin treatment on Δ*xerC* cells, with elevated pyocin expression (Fig. 3C) and, accordingly, poorer growth. This finding raises the possibility that chemical inhibitors of XerC might sensitize P. aeruginosa to fluoroquinolone antibiotics via increased production of pyocins. Notably, recent work indicates that many clinical isolates are sensitive to pyocins and/or produce pyocins themselves (3, 4). Hence, we envision an exciting prospect for combination treatment with a pyocin-stimulating XerC inhibitor plus a fluoroquinolone. P. aeruginosa cells would not only become more antibiotic sensitive but also release increased quantities of P. aeruginosa-killing pyocins as they die, amplifying the bactericidal effect.

## MATERIALS AND METHODS

### Strains and growth conditions.

Escherichia coli SM10 and Pseudomonas aeruginosa PA14 were grown in Luria-Bertani (LB) Lennox broth (10 g/L tryptone, 5 g/L yeast extract, 5 g/L NaCl) or on LB agar plates fortified with 1.5% Bacto agar at 37°C. When appropriate, 25 μg/mL Irgasan (to specifically select for P. aeruginosa) plus 75 μg/mL tetracycline, 25 μg/mL Irgasan plus 75 μg/mL gentamicin, 25 μg/mL tetracycline, or 20 μg/mL gentamicin was added to liquid or solid media. P. aeruginosa was also selected over E. coli for some strains by growth on Vogel-Bonner minimal medium (VBMM) containing citrate as the sole carbon source (48). The strains used in this work are listed in Table 1 and Table S1. Markerless deletions were generated using the pEXG2 vector with counterselection on LB plates containing 6% sucrose or no-salt LB plates containing 15% sucrose (48) and were screened by colony PCR for the presence of deletions. Complementation and reporter strains were constructed by integration of the mini-CTX-1 vector at the neutral chromosomal *attB* locus. Modes of strain and plasmid construction are given in the supplemental material.

**TABLE 1:** Pseudomonas aeruginosa strains used in this study^*a*^

Strain	Genotype or description	Source or reference
PA14 (MTC1)	Laboratory wild-type strain of P. aeruginosa	Laboratory stock; Stephen Lory, Harvard Medical School
MTC590	PA14 *ΔamrZ*	29
MTC1398	PA14 *ΔamrZ Δ69700*	29
MTC1513	PA14 *Δ69700*	29
MTC2191	S13	Pradeep Singh lab (University of Washington)
MTC2192	PML1516d	Pradeep Singh lab (University of Washington)
MTC2193	NIH5	Pradeep Singh lab (University of Washington)
MTC2252	PA14 *ΔxerC attB*::*CTX-1*-P*_07970_*-*gfp*, Tet^r^	This study
MTC2262	PA14 *ΔxerC attB*::*CTX-1*-P*_lppL_*-*xerC*, Tet^r^	This study
MTC2264	PA14 *Δ69700 attB*::*CTX-1*-P*_lppL_*-*xerC*, Tet^r^	This study
MTC2266	PA14 *ΔxerC*	This study
MTC2274	PA14 *ΔrecA*	This study
MTC2276	PA14 *ΔprtN*	This study
MTC2277	PA14 *attB*::*CTX-1*-P*_07970_*-*gfp*, Tet^r^	This study
MTC2280	PA14 *attB*::*CTX-1*-P*_07970_*-*lux*, Tet^r^	This study
MTC2281	PA14 *Δ69700 attB*::*CTX-1*-P*_07970_*-*lux*, Tet^r^	This study
MTC2284	PA14 *Δholin Δlysin attB*::*CTX-1*-P*_07970_*-*lux*, Tet^r^	This study
MTC2285	PA14 *Δ69700 Δholin Δlysin attB*::*CTX-1*-P*_07970_*-*lux*, Tet^r^	This study
MTC2288	PA14 *ΔxerC ΔrecA*	This study
MTC2289	PA14 *ΔxerC ΔprtN*	This study
MTC2291	PA14 *ΔxerC ΔrecA attB*::*CTX-1*-P*_07970_*-*gfp*, Tet^r^	This study
MTC2292	PA14 *ΔxerC ΔprtN attB*::*CTX-1*-P*_07970_*-*gfp*, Tet^r^	This study
MTC2293	PA14 *ΔxerC Δholin Δlysin attB*::*CTX-1*-P*_07970_*-*gfp*, Tet^r^	This study
MTC2294	PA14 *ΔxerC Δholin Δlysin*	This study
MTC2295	PA14 *Δholin Δlysin*	This study
MTC2297	PA14 *ΔxerC attB*::*CTX-1*-P*_07970_*-*lux*, Tet^r^	This study
MTC2298	PA14 *ΔxerC ΔprtN attB*::*CTX-1*-P*_07970_*-lux, Tet^r^	This study
MTC2299	PA14 *ΔxerC Δholin Δlysin attB*::*CTX-1*-P*_07970_*-*lux*, Tet^r^	This study
MTC2301	PA14 *ΔxerC ΔrecA attB*::*CTX-1*-P*_07970_*-lux, Tet^r^	This study
MTC2302	PA14 *ΔrecA attB*::*CTX-1*-P*_07970_*-*lux*, Tet^r^	This study
MTC2304	PA14 *ΔxerC prtR*_S162A_	This study
MTC2305	PA14 *prtR*_S162A_	This study
MTC2307	PA14 *ΔxerC prtR*_S162A_ *attB*::*CTX-1*-P*_07970_*-*lux*, Tet^r^	This study
MTC2308	PA14 *prtR*_S162A_ *attB*::*CTX-1*-P*_07970_*-*lux*, Tet^r^	This study
MTC2324	PA14 Δ*xerC* Δ*pyocins* (Δ*07970-08300*)	This study
MTC2326	PA14 Δ*pyocins* (Δ*07970-08300*)	This study
MTC2332	PA14 Δ*xerC* Δ*pyocins attB*::*CTX-1*-P*_07970_*-*gfp*, Tet^r^	This study
MTC2337	PA14 *xerC*_Y272F_	This study
MTC2339	PA14 *xerC*_Y272F_ *attB*::*CTX-1*-P*_07970_*-*lux*, Tet^r^	This study
MTC2341	PA14 *xerC*_Y272F_ *attB*::*CTX-1*-P*_07970_*-*gfp*, Tet^r^	This study

a E. coli strains, plasmids, oligonucleotides, and modes of strain construction are listed in the supplemental material.

10.1128/mBio.02893-21.3 TABLE S1 Escherichia coli strains used in this study. Download Table S1, DOCX file, 0.02 MB. Copyright © 2021 Baggett et al. 2021 Baggett et al. https://creativecommons.org/licenses/by/4.0/ This content is distributed under the terms of the Creative Commons Attribution 4.0 International license.

### Biofilm assays.

P. aeruginosa biofilm studies were conducted using on solid (1% agar) M6301 medium composed of 100 μM KH_2_PO_4_, 15.14 mM (NH_4_)_2_SO_4_, and 0.36 μM FeSO_4_·H_2_O (pH balanced to 7.0 using 10 M KOH) (49); after autoclaving and before use, 0.5% glycerol, 1 mM MgSO_4,_ and 0.2% Casamino Acids (BD Bacto, USA) were added. Plates containing 40 mL of M6301 with 1% agar were poured fresh for each experiment and allowed to harden for 6 to 7 h. P. aeruginosa cultures grown at 37°C for 6 to 8 h in 3 mL LB were back-diluted to an OD_600_ of 1.0, and 2 μL of the diluted cultures was spotted on M6301 agar plates. The plates were incubated right side up at 25°C and were typically photographed after 4 or 6 days, as indicated.

### RNA isolation and sequencing.

RNA sequencing was achieved by growing the strain of interest in quadruplicate on solid M6301–1% agar plates for 3 days. Total RNA was isolated from homogenized colonies using the New England Biolabs Monarch total RNA miniprep kit. Subsequent quality control steps, the rRNA depletion, Illumina library preparation, and paired-end high-throughput Illumina sequencing were performed by Novogene (Beijing, China). Sequence mapping and analysis were performed at the Oklahoma University Health Sciences Center Laboratory for Molecular Biology and Cytometry Research using CLC software.

### Growth curve analysis.

Strains of interest were grown on LB plates overnight, then inoculated into LB liquid broth until late stationary phase was reached (about 18 h). Strains were then diluted 1,000-fold into fresh medium and grown to early exponential phase (about 4 h). The optical density at 600 nm (OD_600_) was measured, and all cultures were normalized to an OD_600_ equal to 0.1. A 96-well plate containing 160 μL of LB liquid broth containing 0.03 μg/mL ciprofloxacin was inoculated with 20 μL of the normalized cultures. The plate was incubated in a BioTek Synergy H1 plate reader (BioTek, USA) at 37°C for 20 h with orbital shaking. OD_600_ measurements were obtained every 2 min.

### Pyocin indicator assays.

Strains of interest were grown in 10 mL of LB liquid broth at 37°C until stationary phase was reached (about 12 h). OD_600_ measurements were obtained, and the cultures were normalized to the lowest value with a volume of 8 mL in 15-mL centrifuge tubes. The cells were then pelleted by centrifugation (4,500 × *g*, 10 min, 25°C); the supernatants were harvested and filtered using a 0.22-μm syringe filter to remove any remaining cells. Filtered supernatants were stored at 4°C and used within 2 to 3 days. Indicator strains were grown in 3 mL of LB liquid at the same time as the strains of interest. The dense cultures were diluted 1,000-fold into a microcentrifuge tube; then, 150 μL of the diluted cultures was spread plated onto a LB plate using sterile glass beads. The filtered supernatants of the strains of interest were then used undiluted or diluted with sterile LB. Ten microliters of undiluted or diluted supernatants was spotted on top of the indicator strain plates. The plates were then incubated at 37°C overnight.

### Kinetic luciferase assay.

Strains of interest were cultured as described in “Growth curve analysis.” Luminescence was measured in black or white clear-bottom 96-well microtiter plates at 3-min intervals at a sensitivity (gain) setting of 135 or 200 together with the OD_600_ for 20 h on a BioTek Synergy H1 plate reader (BioTek, USA). Final luciferase activity values were calculated by normalizing luciferase luminescence to culture density. Because of differences in the plates (black or white) and gain settings, the luminescence/OD values are not always comparable from graph to graph.

### Fluorescence microscopy.

Strains of interest were grown in 3 mL of LB liquid broth overnight to obtain a saturated culture. They were then diluted 1,000-fold in fresh LB and grown to early exponential phase (about 4 h). Cultures were concentrated by centrifuging 1 mL of exponential culture into a microcentrifuge tube at 5000 *g* for 1 min and resuspending in 100 μL. Cells were immobilized by spotting 0.5 μL of the concentrated mixture onto the pad and covering with cover glass. Imaging was performed on a Nikon Eclipse Ti inverted fluorescence microscope equipped with a Photometrics Prime 95B sCMOS digital camera, a Lumencor SOLA SE II 365 LED Light Engine, and an OKO temperature-controlled enclosure. Snapshot images of the slides were taken at ×100 magnification in both phase and GFP channels. Automated time-lapse imaging was performed at 37°C. For quantification of GFP-positive cells, images were analyzed using the MicrobeJ plugin for ImageJ (50), segmenting on phase-contrast and taking the mean GFP values of the corresponding fluorescence images. Segmentation was performed with default values except that a minimum and maximum areas of 100 and 400 pixels were used, and circularity was delimited from 0 to 0.9. For options, “exclude on edges,” “shape descriptors,” “segmentation,” and “intensity” were selected. A threshold of 1.2 times the average background fluorescence was selected to denote GFP positivity, as 100% of PA14 cells without a GFP reporter fell below this threshold, which was approximately 5.5 standard deviations above the mean fluorescence of reporter-free cells (Fig. S2).

### Quantification of cell debris/vesiculation.

Strains of interest were grown in 10 mL of LB broth at 37°C for 12 h. Once grown, the cultures were equalized to the culture with the lowest OD_600_. The cells were then pelleted by centrifugation (4,500 × *g*, 12 min, 4°C); the supernatants were harvested and filtered using a 0.22-μm syringe filter to remove excess bacteria. The filtrates were then centrifuged again in a TLA100.3 rotor at 80,000 rpm for 30 min at 4°C in a Beckman Coulter TL-100 ultracentrifuge. The supernatants were gently decanted, and the pellets were resuspended in 100 μL phosphate-buffered saline (PBS) supplemented with 0.2 M NaCl. The resuspended material was stained using FM4-64 at a final concentration of 3.3 μg/mL for 10 min at 37°C. Fluorescence was measured using a BioTek Synergy H1 plate reader (BioTek, USA) with an excitation wavelength of 506 nm and an emission wavelength of 700 nm.

10.1128/mBio.02893-21.4 TABLE S2 Plasmids used in this study. Download Table S2, DOCX file, 0.02 MB. Copyright © 2021 Baggett et al. 2021 Baggett et al. https://creativecommons.org/licenses/by/4.0/ This content is distributed under the terms of the Creative Commons Attribution 4.0 International license.

10.1128/mBio.02893-21.5 TABLE S3 Primers used in this study. Download Table S3, DOCX file, 0.02 MB. Copyright © 2021 Baggett et al. 2021 Baggett et al. https://creativecommons.org/licenses/by/4.0/ This content is distributed under the terms of the Creative Commons Attribution 4.0 International license.

10.1128/mBio.02893-21.6 TEXT S1 Modes of strain construction for all P. aeruginosa and E. coli strains reported in this paper, along with the modes of plasmid construction for all plasmids reported in this paper. Download Text S1, DOCX file, 0.03 MB. Copyright © 2021 Baggett et al. 2021 Baggett et al. https://creativecommons.org/licenses/by/4.0/ This content is distributed under the terms of the Creative Commons Attribution 4.0 International license.
