# Supplementary figures and images for: SOS-Independent Pyocin Production in P. aeruginosa Is Induced by XerC Recombinase Deficiency
Source: mBio. 2021 Nov 23;12(6):e02893-21. doi: 10.1128/mBio.02893-21 (PMC8609362; doi:10.1128/mBio.02893-21)

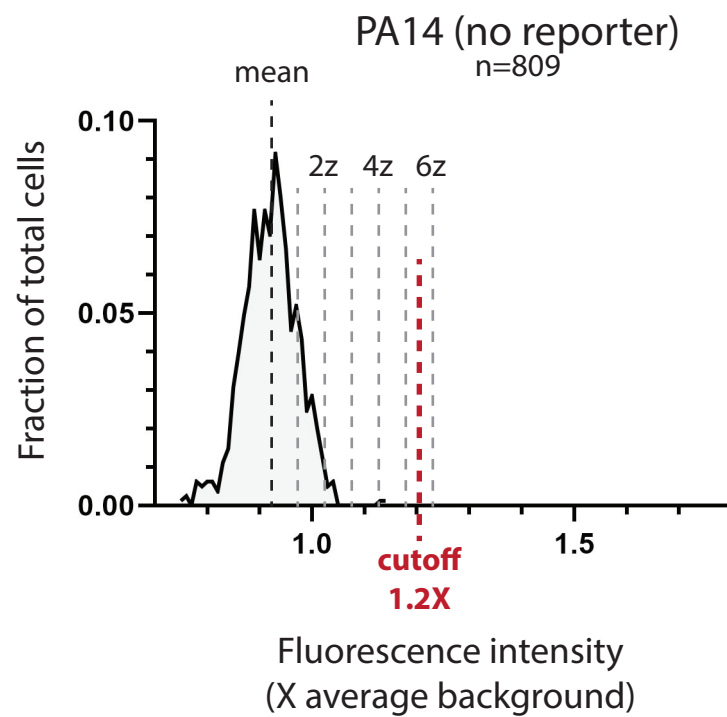

Figure S2

Supplement: FIG S2 [file mbio.02893-21-sf002.pdf]

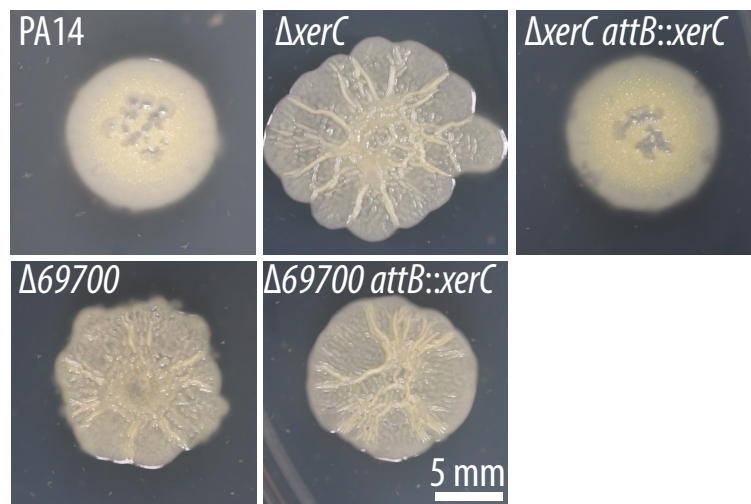

Figure S1

Supplement: FIG S1 [file mbio.02893-21-sf001.pdf]
