# Supplementary material for: SOS-Independent Pyocin Production in P. aeruginosa Is Induced by XerC Recombinase Deficiency
Source: mBio. 2021 Nov 23;12(6):e02893-21. doi: 10.1128/mBio.02893-21 (PMC8609362; doi:10.1128/mBio.02893-21)
Supplement: TABLE S1 [file mbio.02893-21-st001.docx]

**Baggett, Bronson *et al.* | Supplementary Information**

**Table S1. *Escherichia coli* strains used in this study.**

| MTC27 | SM10 (*F- endA1 hsdR17 supE44 thi-1 λ- recA1 gyrA96 relA1*); *E. coli* mating strain for conjugation with *P. aeruginosa* | (1) |
| --- | --- | --- |
| MTC570 | SM10 pEXG2-*∆amrZ*, Gent^R^ | (2) |
| MTC1346 | SM10 pEXG2-*∆69700*, Gent^R^ | (2) |
| MTC2163 | SM10 pEXG2-∆*07970-08300* (∆*pyocin*) | This study |
| MTC2176 | SM10 pEXG2-*∆prtN*, Gent^R^ | This study |
| MTC2179 | SM10 pEXG2-*∆recA*, Gent^R^ | This study |
| MTC2212 | SM10 pEXG2-*∆xerC*, Gent^R^ | This study |
| MTC2220 | SM10 pEXG2-*∆lysin*, Gent^R^ | This study |
| MTC2226 | SM10 pEXG2-*∆holin*, Gent^R^ | This study |
| MTC2261 | SM10 pCTX-1-P*_lppL_*-*xerC*, Tet^R^ | This study |
| MTC2286 | SM10 pCTX-1-P*_07970_*-gfp, Tet^R^ | This study |
| MTC2287 | SM10 pCTX-1-P*_07970_*-lux, Tet^R^ | This study |
| MTC2306 | SM10 pEXG2-*prtR*_S162A_, Gent^R^ | This study |
| MTC2334 | SM10 pEXG2-*xerC_Y272F_* | This study |

**References**

1. Simon R, Priefer U, Pühler A. 1983. A Broad Host Range Mobilization System for In Vivo Genetic Engineering: Transposon Mutagenesis in Gram Negative Bacteria. Bio/Technology 1:784-791.

2. Cabeen MT, Leiman SA, Losick R. 2016. Colony-morphology screening uncovers a role for the Pseudomonas aeruginosa nitrogen-related phosphotransferase system in biofilm formation. Mol Microbiol 99:557-70.
