# Supplementary material for: SOS-Independent Pyocin Production in P. aeruginosa Is Induced by XerC Recombinase Deficiency
Source: mBio. 2021 Nov 23;12(6):e02893-21. doi: 10.1128/mBio.02893-21 (PMC8609362; doi:10.1128/mBio.02893-21)
Supplement: TABLE S2 [file mbio.02893-21-st002.docx]

**Baggett, Bronson *et al.* | Supplementary Information**

**Table S2. Plasmids used in this study.**

| **Plasmid** | **Description** | **Source or reference** |
| --- | --- | --- |
| pCTX-1 | mini-CTX-1, integrative tet^R^ plasmid for *P. aeruginosa* | (1) |
| pCTX-1-lux | CTX-1 reporter vector containing *luxA-E* | (2) |
| pCTX-1-gfp | CTX-1 reporter vector containing *gfp* | (1) |
| pCTX-1-P*_07970_*-lux | CTX-1 lux reporter driven from *PA14_07970* promoter | This study |
| pCTX-1-P*_07970_*-gfp | CTX-1 gfp reporter driven from *PA14_07970* promoter | This study |
| pEXG2 | Integrating suicide plasmid for *P. aeruginosa*, gent^R^, with sucrose counterselection | (3) |
| pEXG2-∆*prtN* | EXG2 containing flanking sequences of *prtN* | This study |
| pEXG2-∆*recA* | EXG2 containing flanking sequences of *recA* | This study |
| pEXG2-∆*xerC* | EXG2 containing flanking sequences of *xerC* | This study |
| pEXG2-∆*holin* | EXG2 containing flanking sequences of *holin* | This study |
| pCTX-1-P*_lppL_*-*xerC* | CTX-1 containing fusion of promoter of *lppL* (operon promoter) stitched with *xerC* coding sequence | This study |
| pEXG2-∆*lysin* | EXG2 containing flanking sequences of *lysin* | This study |
| pEXG2-*prtR*_S162A_ | EXG2 containing sequence encoding a mutation at the PrtR autoproteolytic residue (162) from serine to alanine | This study |
| pEXG2-∆*07970-08300* (pEXG2-∆*pyocins*) | EXG2 containing flanking sequences of the R/F pyocin cluster, running from *PA14_07970* to *PA14_08300* | This study |
| pEXG2-*xerC_Y272F_* | EXG2 containing sequence encoding a mutation at the XerC active-site Tyr residue (272) to phenylalanine | This study |

**References**

1. Hoang TT, Kutchma AJ, Becher A, Schweizer HP. 2000. Integration-proficient plasmids for Pseudomonas aeruginosa: site-specific integration and use for engineering of reporter and expression strains. Plasmid 43:59-72.

2. Becher A, Schweizer HP. 2000. Integration-proficient Pseudomonas aeruginosa vectors for isolation of single-copy chromosomal lacZ and lux gene fusions. Biotechniques 29:948-50, 952.

3. Rietsch A, Vallet-Gely I, Dove SL, Mekalanos JJ. 2005. ExsE, a secreted regulator of type III secretion genes in Pseudomonas aeruginosa. Proc Natl Acad Sci U S A 102:8006-11.
