# Supplementary material for: SOS-Independent Pyocin Production in P. aeruginosa Is Induced by XerC Recombinase Deficiency
Source: mBio. 2021 Nov 23;12(6):e02893-21. doi: 10.1128/mBio.02893-21 (PMC8609362; doi:10.1128/mBio.02893-21)
Supplement: TABLE S3 [file mbio.02893-21-st003.docx]

**Baggett, Bronson *et al.* | Supplementary Information**

**Table S3. Primers used in this study.**

| **Primer Name or No.** | **Sequence (5'-3')** |
| --- | --- |
| 630 | **GACGGTATCGATAAGCTTGATATC GAATTC** TCTCCAGGTCGAGATCCAG |
| 902 | **TTATACGAGCCGGAAGCATAAATGTAAAGC AAGCT** GGAAATAGTCAAACGTAGCCTGCAC |
| 1047 | **TGCGCACCCGTGGAAATTAATTAAGGTACC GAATTC** GCTGTTGCCGGTGAGTTGG |
| 1048 | **AGACCGACCG** GTGAAATCTCTGAAAGGCGGG |
| 1049 | **GAGATTTCAC** CGGTCGGTCTGAGAGTTCGTG |
| 1074 | **GCTGTCCGCG** GGTATTCCCTCCTGCGGCTG |
| 1075 | **TGCGCACCCGTGGAAATTAATTAAGGTACC GAATTC** CGCTGCATCCGTGGGACAG |
| 1076 | **AGGGAATACC** CGCGGACAGCATCGCATCC |
| 1077 | **TTATACGAGCCGGAAGCATAAATGTAAAGC AAGCTT** CATCGGCGCGGTGAAGGTC |
| 1078 | **GACGGTATCGATAAGCTTGATATC GAATTC** AGCAAGGGCGAGGTGGTCC |
| 1079 | **TTTACCGGCAGATTTCTAAAGAAGAATTGG GGATCC** GAGTGCCTCCCTGGGGACG |
| 1084 | **TGCGCACCCGTGGAAATTAATTAAGGTACC GAATTC** CCATGTGAAATCTCTGAAAGGCGGG |
| 1085 | CGGTTCCATGGCGTTGCCGGTGAGTTGGGC |
| 1086 | CGGCAACGCCATGGAACCGCTGATCATGG |
| 1087 | **TTATACGAGCCGGAAGCATAAATGTAAAGC AAGCTT** CGGCGAGGTGATCGAGGGC |
| 1122 | **TCTAGAACTAGTGGATCCCCCGGGCTGCAG GAATTC** GGTTGGGTCTTCTGGTGGTCC |
| 1123 | **CCCCCTCGAGGTCGACGGTATCGAT AAGCTT** GCTCCCTACGATGCTGGCAAC |
| 1140 | **TGCGCACCCGTGGAAATTAATTAAGGTACC GAATTC** CAACATCGAGATGCGCCACC |
| 1141 | **CGCCCGTGGC** GTCGGCGCGCATCTCGGAC |
| 1142 | **CGCCCGTGGC** GTCGGCGCGCTTCTCGGAC |
| 1143 | **GCGCGCCGAC** GCCACGGGCGATCCAACTG |
| 1144 | **TTATACGAGCCGGAAGCATAAATGTAAAGC AAGCTT** GAGGATCTCCAGGGTCGGGTG |
| 1165 | **TGCGCACCCGTGGAAATTAATTAAGGTACC GAATTC** GCAAGGGCGAGGTGGTCCGC |
| 1166 | **CACGGTCGCC** GGCCGGGTTCCGGTGCTTCAC |
| 1167 | **GAACCCGGCC** GGCGACCGTGGCCCCCATTG |
| 1168 | **TTATACGAGCCGGAAGCATAAATGTAAAGC AAGCTT** CACGGCGACCACCACGCAGG |
| 1169 | **TGCGCACCCGTGGAAATTAATTAAGGTACC GAATTC** GGTCAATGAGTCCGACTACCAC |
| 1170 | **CCCTGGCCCG** CAATTGCTGCTCGGTCAGTTTC |
| 1171 | **GCAGCAATTG** CGGGCCAGGGCGGTGCTG |
| 1172 | **TTATACGAGCCGGAAGCATAAATGTAAAGC AAGCTT** GTGTCTCAGCGGCAGGCAC |
| 1173 | **TTTACTCATATGTATATCTCCTTCTTAAATCTAGA GGATCC** GAGTGCCTCCCTGGGGACG |
| 1222 | **TGCGCACCCGTGGAAATTAATTAAGGTACC GAATTC** CCAGAAACAGGGCGCCACC |
| 1223 | **ACTTGGCGGC** GTCCGCCTTGCCCTGGC |
| 1224 | **CAAGGCGGAC** GCCGCCAAGTACCTGGAAGAC |
| 1225 | **TTATACGAGCCGGAAGCATAAATGTAAAGC AAGCTT** CTGCCGGAAAACTGGGACTGAAG |
| 1273 | **CGGCGCGCAT** GACGGAATCCTTGTAGAAAACG |
| 1274 | **GGATTCCGTC** ATGCGCGCCGACCTGGATGC |
| 1275 | **CCCCCTCGAGGTCGACGGTATCGAT AAGCTT** CCACAGGGTGTCGTCGAGGTCG |
| 1291 | **TGCGCACCCGTGGAAATTAATTAAGGTACC GAATTC** GGGGAATGGGCTCGCCGCAG |
| 1292 | **CACCAGGGAC** TCGGGCGGGATCTGGGTGCTC |
| 1293 | **TCCCGCCCGA** GTCCCTGGTGCGGTGAGCGC |
| 1294 | **TTATACGAGCCGGAAGCATAAATGTAAAGC AAGCTT** GGCGGATGGCGTTTGCGTCATC |
| 1331 | **GACGGTATCGATAAGCTTGATATC GAATTC GTCCCTGGTGCGGTGAGCGC** |
| 1332 | **TTTACCGGCAGATTTCTAAAGAAGAATTGG GGATCC GGTATTCCCTCCTGCGGCTG** |
| 1337 | TGATCAGCGGTTCCATGGC |
| 1338 | TGATCAGCGGTTCCATGCT |
| 1407 | AGGTGGGTGAAGATCTGGGTCGTG |
| 1408 | GACCCAGATCTTCACCCACCTGGAC |

NB: The listed primer sequences may include 5' overlaps for isothermal assembly and/or stitch PCR (in boldface type). The 3' end is complementary to the target genomic sequence
