# Supplementary material for: SOS-Independent Pyocin Production in P. aeruginosa Is Induced by XerC Recombinase Deficiency
Source: mBio. 2021 Nov 23;12(6):e02893-21. doi: 10.1128/mBio.02893-21 (PMC8609362; doi:10.1128/mBio.02893-21)
Supplement: TEXT S1 [file mbio.02893-21-t0001.docx]

**Baggett, Bronson *et al.* | Supplementary Information**

**Modes of strain construction**

***P. aeruginosa* strains**

**MTC2219**

MTC1 was mated with MTC2220: 50 µL of an overnight LB culture of MTC2220 was spot-dried on an LB plate, and 100 µL of an overnight LB culture of MTC1 was subsequently dried on top of the first spot. The plate was incubated at 37°C overnight, and the resulting colony was scraped up with a sterile loop and resuspended in 500 µL sterile phosphate-buffered saline (PBS). An aliquot (typically 100 µL) of the suspension was spread on LB agar containing 75 µg/ml gentamycin and 25 µg/ml irgasan to select for *P. aeruginosa* transformants with integrated pEXG2-*Δlysin* (∆*PA14_08160*) plasmid. Several of the resulting colonies were then inoculated into plain LB and grown at 37°C for 4-6 hours to accumulate second crossovers. Aliquots (typically 25 µL and 100 µL) of the LB culture were then spread on LB plates containing 6% sucrose to select against the plasmid. A number of the sucrose-resistant colonies arising were then patched on LB and LB with 20 µg/mL gentamycin. At least 2 sucrose-resistant, gent-sensitive clones were then streaked for single colonies, checked by PCR for presence of the desired deletion, and frozen at -80°C in 25% glycerol.

For strains MTC2324 and higher numbers, after mating, transformants were selected on VBMM with 25 µg/ml gentamycin and then re-streaked on no-salt LB with 15% sucrose to select second crossovers, as in Hmelo *et al*. (1). A number of the sucrose-resistant colonies arising were then patched on LB and LB with 20 µg/mL gentamycin. At least 2 sucrose-resistant, gent-sensitive clones were then streaked for single colonies, checked by PCR and/or sequencing for presence of the desired deletion, and frozen at -80°C in 25% glycerol.

**MTC2252**

MTC2266 was mated with MTC2286 as described above for MTC2219, and a 10-µL aliquot of the PBS-resuspended mating mix was spread on LB plates with 75 µg/mL tetracycline and 25 µg/mL irgasan to select for *P. aeruginosa* transformants. At least 2 colonies were then re-streaked for single colonies on LB-tet (25 µg/mL), grown in LB overnight at 37°C, and stored in 25% glycerol at -80°C.

For strains MTC2332 and higher numbers, after mating, *P. aeruginosa* transformants were selected on VBMM with 25 µg/ml tetracycline and then re-streaked for single colonies on LB-tet (25 µg/mL), following the protocol of Hmelo *et al.* (1).

**MTC2262**

Constructed like MTC2252, but MTC2266 was mated with MTC2261.

**MTC2264**

Constructed like MTC2252, but MTC1513 was mated with MTC2261.

**MTC2266**

Constructed like MTC2219, but MTC1 was mated with MTC2212.

**MTC2274**

Constructed like MTC2219, but MTC1 was mated with MTC2179.

**MTC2276**

Constructed like MTC2219, but MTC1 was mated with MTC2176.

**MTC2277**

Constructed like MTC2252, but MTC1was mated with MTC2286.

**MTC2280**

Constructed like MTC2252, but MTC1 was mated with MTC2287.

**MTC2281**

Constructed like MTC2252, but MTC1513 was mated with MTC2287

**MTC2284**

Constructed like MTC2252, but MTC2295 was mated with MTC2287.

**MTC2288**

Constructed like MTC2219, but MTC2266 was mated with MTC2179.

**MTC2289**

Constructed like MTC2219, but MTC2266 was mated with MTC2176.

**MTC2291**

Constructed like MTC2252, but MTC2288 was mated with MTC2286.

**MTC2292**

Constructed like MTC2252, but MTC2289 was mated with MTC2286.

**MTC2293**

Constructed like MTC2252, but MTC2294 was mated with MTC2286.

**MTC2294**

Constructed like MTC2219, but MTC2295 was mated with MTC2212.

**MTC2295**

Constructed like MTC2219, but MTC590 was mated with MTC2226.

**MTC2297**

Constructed like MTC2252, but MTC2266 was mated with MTC2287.

**MTC2298**

Constructed like MTC2252, but MTC2289 was mated with MTC2287.

**MTC2299**

Constructed like MTC2252, but MTC2294 was mated with MTC2287.

**MTC2301**

Constructed like MTC2252, but MTC2288 was mated with MTC2287.

**MTC2302**

Constructed like MTC2219, but MTC2274 was mated with MTC2287.

**MTC2304**

Constructed like MTC2219, but MTC2266 was mated with MTC2306.

**MTC2305**

Constructed like MTC2219, but MTC1 was mated with MTC2306.

**MTC2307**

Constructed like MTC2252, but MTC2304 was mated with MTC2287.

**MTC2308**

Constructed like MTC2252, but MTC2305 was mated with MTC2287.

**MTC2324**

Constructed like MTC2219, but MTC2266 was mated with MTC2163.

**MTC2326**

Constructed like MTC2219, but MTC1 was mated with MTC2163.

**MTC2332**

Constructed like MTC2252, but MTC2326 was mated with MTC2286.

**MTC2337**

Constructed like MTC2219, but MTC1 was mated with MTC2334.

**MTC2339**

Constructed like MTC2252, but MTC2336 was mated with MTC2287.

**MTC2341**

Constructed like MTC2252, but MTC2336 was mated with MTC2286.

***E. coli* strains**

**MTC2163, 2176, 2179, 2212, 2220, 2226, 2306, 2334**

The appropriate pEXG2-derived knockout plasmids (listed in Table S2) were electroporated into SM10 (MTC27), and transformants were selected on LB plates containing 20 µg/mL gentamycin.

**MTC2261, 2286, 2287**

The appropriate mini-CTX-1 or mini-CTX-1-lux derivatives (listed in Table S2) were electroporated into SM10 (MTC27), and transformants were selected on LB plates containing 25 µg/mL tetracycline.

**Modes of plasmid construction**

All plasmids constructed in this study were assembled from purified PCR products (using the primers listed in Table S3) and restriction enzyme-cleaved plasmid backbones by using isothermal assembly (2). The insert sequences of all plasmids were verified via Sanger sequencing before they were used to create new strains.

**pCTX-1-P*_07970_*-lux**

The promoter of the first gene of the pyocin region (*PA14_07970*) was PCR-amplified from PA14 genomic DNA using primers 1078 and 1079 and assembled into EcoRI/BamHI-cleaved pCTX-1-lux.

**pCTX-1-P*_07970_*-gfp**

The promoter of the first gene of the pyocin region (*PA14_07970*) was PCR-amplified from PA14 genomic DNA using primers 1078 and 1173 and assembled into EcoRI/BamHI-cleaved pCTX-1-gfp.

**pEXG2-Δ*prtN***

The upstream and downstream flanking sequences of the *prtN* coding sequence were amplified from PA14 chromosomal DNA using primer pairs 1074/1075 and 1076/1077, respectively. A fragment containing the *prtN* gene deletion was generated by stitch PCR using the initial fragments as self-priming templates with primers 1075 and 1077. The resulting deletion fragment was assembled into EcoRI/HindIII-cleaved pEXG2.

**pEXG2-Δ*recA***

The upstream and downstream flanking sequences of the *recA* coding sequence were amplified from PA14 chromosomal DNA using primer pairs 986/987 and 988/989, respectively. A fragment containing the *recA* gene deletion was generated by stitch PCR using the initial fragments as self-priming templates with primers 986 and 989. The resulting deletion fragment was assembled into EcoRI/HindIII-cleaved pEXG2.

**pEXG2-Δ*xerC***

The upstream and downstream flanking sequences of the *xerC* coding sequence were amplified from PA14 chromosomal DNA using primer pairs 1140/1141 and 1143/1144, respectively. A fragment containing the *xerC* gene deletion was generated by stitch PCR using the initial fragments as self-priming templates with primers 1140 and 1144. The resulting deletion fragment was assembled into EcoRI/HindIII-cleaved pEXG2.

**pEXG2-Δ*holin***

The upstream and downstream flanking sequences of the *PA14_07990* (encoding the holin enzyme) coding sequence were amplified from PA14 chromosomal DNA using primer pairs 1165/1166 and 1167/1168, respectively. A fragment containing the *holin* gene deletion was generated by stitch PCR using the initial fragments as self-priming templates with primers 1165 and 1168. The resulting deletion fragment was assembled into EcoRI/HindIII-cleaved pEXG2.

**pCTX-1-P*_lppL-xerC_***

The presumed *lppL* promoter driving the operon that includes *69700* and *xerC* was amplified with primers 630 and 1273. This fragment was then stitched via self-priming PCR (using primers 630 and 1275) to the *xerC* coding sequence, which was amplified with primers 1274 and 1275. The resulting fragment was assembled into EcoRI/HindIII-cleaved pCTX-1.

**pEXG2-Δ*lysin***

The upstream and downstream flanking sequences of the *PA14_08160* (encoding the lysin enzyme) coding sequence were amplified from PA14 chromosomal DNA using primer pairs 1169/1170 and 1171/1172, respectively. A fragment containing the *lysin* gene deletion was generated by stitch PCR using the initial fragments as self-priming templates with primers 1169 and 1172. The resulting deletion fragment was assembled into EcoRI/HindIII-cleaved pEXG2.

**pEXG2-*prtR*_S162A_**

A version of *prtR* encoding the S162A substitution in PrtR and ~600 bp of flanking sequence on either side of the substitution was generated via mutagenic PCR using primers 1084/1085 for the upstream flank and 1086/1087 for the downstream flank. The full fragment was generated by stitch PCR using the initial fragments as self-priming templates with primers 1084 and 1087. The resulting *prtR_S162A_* fragment was assembled into EcoRI/HindIII-cleaved pEXG2.

**pEXG2-∆*07970-08300***

The upstream flanking sequence of the *PA14_07970* (encoding the first gene in the R/F pyocin gene cluster) coding sequence and the downstream flanking sequence of *PA14_08300* (encoding the last gene in the cluster) were amplified from PA14 chromosomal DNA using primer pairs 1047/1048 and 1049/902, respectively. A fragment containing the pyocin gene cluster deletion was generated by stitch PCR using the initial fragments as self-priming templates with primers 1047 and 902. The resulting deletion fragment was assembled into EcoRI/HindIII-cleaved pEXG2.

**pEXG2-*xerC_Y272F_***

A version of *xerC* encoding the Y272F substitution in XerC and flanking sequences (400-800 bp) on either side of the substitution was generated via mutagenic PCR using primers 1140/1407 for the upstream flank and 1408/1144 for the downstream flank. The full fragment was generated by stitch PCR using the initial fragments as self-priming templates with primers 1140 and 1144. The resulting *xerC_Y272F_* fragment was assembled into EcoRI/HindIII-cleaved pEXG2.

**References**

1. Hmelo LR, Borlee BR, Almblad H, Love ME, Randall TE, Tseng BS, Lin C, Irie Y, Storek KM, Yang JJ, Siehnel RJ, Howell PL, Singh PK, Tolker-Nielsen T, Parsek MR, Schweizer HP, Harrison JJ. 2015. Precision-engineering the Pseudomonas aeruginosa genome with two-step allelic exchange. Nat Protoc 10:1820-41.

2. Gibson DG, Young L, Chuang RY, Venter JC, Hutchison CA, 3rd, Smith HO. 2009. Enzymatic assembly of DNA molecules up to several hundred kilobases. Nat Methods 6:343-5.
